# Increased *P16* DNA Methylation in Mouse Thymic Lymphoma Induced by Irradiation

**DOI:** 10.1371/journal.pone.0093850

**Published:** 2014-04-18

**Authors:** Wengang Song, Yongzhe Liu, Ying Liu, Cong Zhang, Bao Yuan, Lianbo Zhang, Shilong Sun

**Affiliations:** 1 Beihua University, Jinlin, China; 2 Department of Toxicology, School of Public Health, Tianjin Medical University, Tianjin, China; 3 Department of Toxicology, School of Public Health, Jilin University, Changchun, China; 4 Ministry of Health, Key Laboratory of Radiobiology, Jilin University, Changchun, China, National Laboratory of Medical Molecular Biology, Tsinghua University, Beijing, PR China; 5 College of Animal Sciences, Jilin University, Changchun, China; 6 Department of Plastic and Reconstructive Surgery, China-Japan Union Hospital of Jilin University, Changchun, China; Peking University Cancer Hospital and Institute, China

## Abstract

DNA methylation is an important part of epigenetics. In this study, we examined the methylation state of two CpG islands in the promoter of the *p16* gene in radiation-induced thymic lymphoma samples. The mRNA and protein levels of P16 were significantly reduced in radiation-induced thymic lymphoma tissue samples. Twenty-three CpG sites of the CpG islands in the *p16* promoter region were detected, and the methylation percentages of −71, −63, −239, −29, −38, −40, −23, 46 CpG sites were significantly higher in radiation-induced thymic lymphoma tissue samples than those in matched non-irradiated thymus tissue samples. This study provides new evidence for the methylation state of *p16* in the radiation-induced thymic lymphoma samples, which suggests that the methylation of these CpG sites in the *p16* promoter may reduce its expression in the thymic lymphoma after irradiation.

## Introduction

Radiation, as a definite factor in carcinogenesis, has been widely investigated and studied, but the epigenetic regulation mechanisms of many radiation sensitive genes have not been clearly explored. The tumor suppressor gene *p16* (also known as CDKN2, INK4a, p14ARF, p19Arf, p16INK4, p16INK4a, and CDKN2A), located on chromosome 9p21, is the most commonly altered gene in human malignancies [1]. The *p16* gene encoding 148 amino acid protein contains 4 ankyrin repeats, and is a cell cycle regulator through inhibiting the G_1_-S phase transition [2]. The *p16* (INK4a) protein acts as a cyclin-dependent kinase inhibitor (CDKI) that impedes the mitosis at G_1_-S transition by inactivation of specific cyclin-protein kinase complexes including cyclin D1, CDK4, and CDK6. *p16* functions as an important tumor suppressor. The loss of *p16* activity through homologous deletion, point mutation, negative regulation of MicroRNAs (miRNAs) or methylation-induced promoter silencing, is a common step in tumor development and progression, which has been widely observed in cancer cell lines and many malignant tumors [3], including acute lymphoblastic leukemia, melanoma, pancreatic cancer, esophagus cancer, lung cancer, bladder cancer, and cervical cancer [4]–[7].

Cytosine methylation is a common form of epigenetic mechanism. CpG sites are the DNA regions where a cytosine nucleotide occurs next to a guanine nucleotide in the linear sequence of bases along its length. Cytosines in CpG dinucleotides can be methylated by DNA methyltransferases to form 5-methylcytosine. The regions in the genome with a higher concentration of CpG sites are named as CpG islands [8]. Many genes have CpG islands in their promoter regions from the upstream of the transcription start site to within the first exon [9]. DNA methylation in the promoter regions is associated with the down-regulation of some genes [10]–[12]. Many transcription factor binding sites have CpG dinucleotides, and methylation of these sites alters their binding to cognate transcription factors [11]–[13]. Methylation of CpG islands around transcription start sites (TSS) represses gene expression epigenetically and plays crucial roles in cell differentiation, development and pathogenesis [14]. However, the mechanistic insights for the role of DNA methylation in carcinogenesis are still unknown. Attempts to analyze the aberrant methylation and its extension patterns within CpG islands in carcinogenesis, especially in the radiation-induced carcinogenesis, have not been successfully achieved. Aberrant methylation of CpG islands is the main reason for *p16* inactivation in multiple human cancers. Aberrant *p16* methylation is an early event in carcinogenesis and has been shown to significantly increase the risk of malignant transformation of epithelial dysplasia in the stomach, oral cavity and other organs in many studies [15]–[17]. Although *p16* methylation is one of the well-studied epigenetic events [18]–[20], most of studies employed in vitro cell culture system. The methylation is not often detectable in tissue samples, especially in the radiation-induced carcinogenesis. In the present study, we studied the relationship between the natural methylation signatures of *p16* CpG islands in the *p16* gene promoter region (from 400 nucleotides upstream to 200 nucleotides downstream of the TSS) and thymic lymphomas.

## Materials and Methods

### Subjects and irradiation

**Figure 1:**
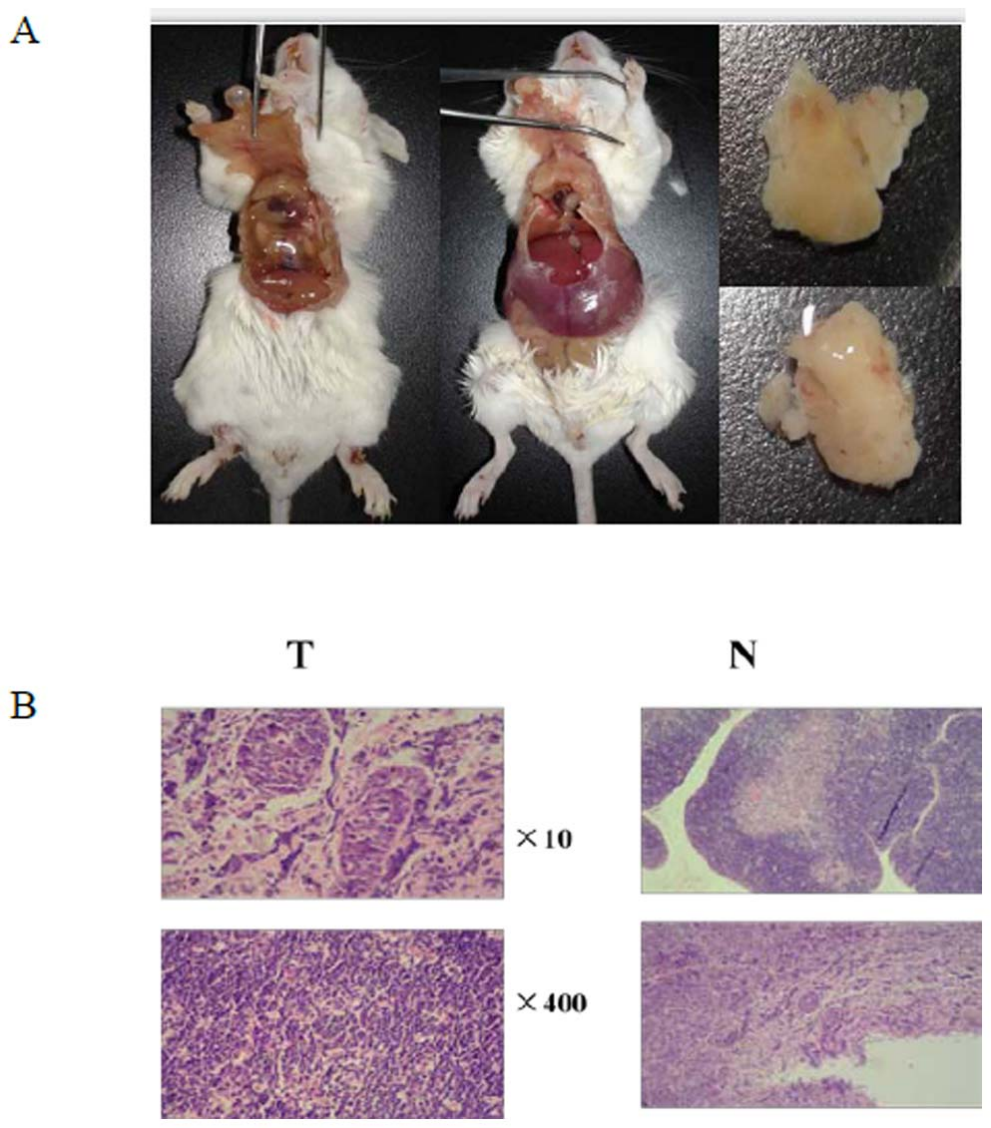
A. Gross image of a thymic lymphoma in a dissected mouse. B. Histological section of a thymic lymphoma stained with Hematoxylin and Eosin (H&E). N: normal control non-irradiated thymus tissue samples. T: radiation induced thymic lymphoma tissue samples.

Male wild-type BALB/c mice, 5–6 weeks of age, were purchased from Jilin university (Changchun, China). All mice were housed in a pathogen-free facility for all experiments. All efforts were made to minimize the number of animals used as well as their suffering. This study was carried out in strict accordance with the recommendations in the Guide for the Care and Use of Laboratory Animals of the National Institutes of Health. The protocol was approved by the Committee on the Ethics of Animal Experiments of the Jilin university (Permit Number: 2011034). All surgery was performed under sodium pentobarbital anesthesia, and all efforts were made to minimize suffering. A fraction of the mice (about 100) were left as controls. Other mice (about 100) were subjected to whole body irradiation split into four weekly sub-lethal doses of 1.75 Gy (Dose rate: 0.58 Gy/min) using a 4-MV linear accelerator (Clinac 4/100, Varian, Palo Alto, CA). Thymic lymphomas were observed to develop at regular intervals of up to 6 months and we find that in our experimental conditions, a global incidence of 43.51% was observed after 6 month (Figure 1A). Thymic lymphoma in mice has been extensively studied as a model of radiation induced carcinogenesis since it was first described by Kaplan et al. in 1953 [21]. Immunological markers and morphologic criteria of thymic lymphoma induced by irradiation have also been commonly described in many studies [22], [23]. In our study, histopathology of thymic lymphoma showed that Thymus inherent structure was replaced by diffuse proliferative lymphoma cells compared with the normal thymus in our study (Figure 1B).

### mRNA expression profile analysis by Illumina Bead Array and quantitative RT-PCR analysis

Total RNA was extracted from the thymic lymphoma tissue samples or thymus tissue samples using the TRIzol method according to the manufacturer's protocol. Labeling was achieved by use of the incorporation of biotin-16-UTP (Perkin Elmer Life and Analytical Sciences, Boston, MA) present at a ratio of 1∶1 with unlabeled UTP. Labeled, amplified material (700 ng per array) was hybridized to a pilot version of the Illumina Sentrix Mouse-6 Expression BeadChip according to the Manufacturer's instructions (Illumina, Inc., San Diego, CA). Amersham fluorolink streptavidin-Cy3 (GE Healthcare Bio-Sciences, Little Chalfont, UK) following the BeadChip manual. Arrays were scanned with an Illumina Bead array Reader confocal scanner according to the Manufacturer's instructions. Array data processing and analysis were performed using Illumina Bead Studio software. More information can be obtained from the Web: http://www.illumina.com/.Total RNA was reverse transcribed to cDNA using Superscript II reverse transcriptase (Invitrogen) and oligonucleotide primers. Quantitative real-time PCR (RT-PCR) analysis of gene expression was performed in a 25 µl reaction volume containing cDNA, SYBR Premix Ex Taq (Takara Bio Inc., Shiga, Japan), TaqMan Universal PCR Master Mixture and primers for each gene.The primers for the *p16* gene were as follows: 5′-GCAGATTCGAACTGCGAG-3′ (forward) and 5′-CACGATGTCTTGATGTCCC-3′ (reverse); The β-actin was used as a reference gene and its PCR primers included: 5′-ATATCGCTGCGCTGGTCGTC-3′ (forward) and 5′- AGGAGTCCTTCTGACCCATTC -3′ (reverse).The relative quantitative method was used for the quantitative analysis and fold change (FC) was used to present data.

### Western blotting

Thymic lymphoma tissue samples induced by irradiation were harvested and lysed after 6 month; proteins were separated on a SDS/polyacrylamide gel and transferred into a PVDF membrane (Bio-Rad, Hercules, CA). After blocking, the membranes were incubated with the primary antibody, anti-P16 polyclonal antibody (Santa Cruz Biotechnology, Santa Cruz, CA). The membranes were extensively washed and incubated with a horseradish peroxidase conjugated secondary antibody (Bio-Rad). The antigen antibody complexes were visualized by West-Q-Chemiluminescent Sub Kit Plus (BIOTANG, Waltham, MA).

### Isolation and bisulfite treatmentof thymus DNA

DNA was extracted from BALB/c mouse thymus using the Gentra Puregene kit (Qiagen GmbH, Hilden, Germany) according to the manufacturer's protocol. Determination of Percent Methylated Cytosine Genomic DNA (300 ng) was treated with sodium bisulfate using the EZ-96 DNA Methylation Kit D5004 (Zymo Research, Orange, CA) according to the manufacturer's protocol. The final bisulfite-treated DNA was eluted in 40 ml M-Elution Buffer. Primers for amplifying the upstream *p16* CpG island using bisulfite-treated DNA were designed using Methyl Primer Express v1.0 (Applied Biosystems, Foster City, CA) and Vector NTI Advance 10 (Invitrogen, Carlsbad, CA). Amplification was performed with 1 µl bisulfite-treated DNA, 1 µM of each primer (primer : 5′-GTTAAAGGGTGATTAGGTATGG-3′ and :5′-ACCCCAACTTCCAACAATAC- 3′, 250 µM each of dATP, dCTP, dGTP, and TTP, 50 µM KCl, 4 µM MgCl2, 0.625 U AmpliTaq Gold (Applied Biosystems), and 10 µM Tris-HCl (pH 8.3) in 50 µl. Amplification consisted of 5 min at 95°C, 40 cycles of 15 sat 95°C, 15 s at 52°C, and 30 s at 72°C, followed by a final elongation step at 72°C for 7 min.

### Cloning of PCR Fragments and Sequencing

Amplified DNA fragments, 474 bp in size, were cloned using the TA Cloning Kit into the pCRII plasmid (Invitrogen).The amplicons were cloned into the PCR-blunt vector, transformed into E. coli, and sequenced using an ABI 3730 Analyzer. At least 6clones were then randomly selected and sequenced for each sample. To demonstrate the initiation and extension of de novo methylation of *p16* CpG islands, the methylation status of each clone was analyzed as a independent variable. There are23 tested CpG sites in the promoter region of *p16* gene.

### Sequence Analysis

The amplified region of *p16* was analyzed for predicted transcription factor-binding sites using Transcription Element Search System (TESS; Schug and Overton, 1977).

### Statistical analysis

All the statistical tests were performed using the SPSS for Windows 11.0 software package (SPSS, Chicago, IL,USA), including descriptive statistics, t- test, the Chi-square test and one-way analysis of variance (ANOVA). The significant level was set at a P-value of <0.05.

## Result

### *p16* is down-regulated in radiation-induced thymic lymphoma tissues

**Figure 2:**
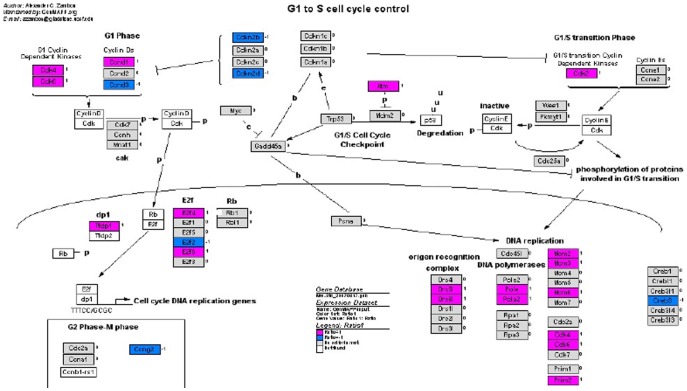
Analysis of mRNA expression profile by the Illumina Sentrix Mouse-6 Expression BeadChip in G1/S cell cycle. Blue: expression down-regulated; Pink: expression up-regulated.

**Figure 3:**
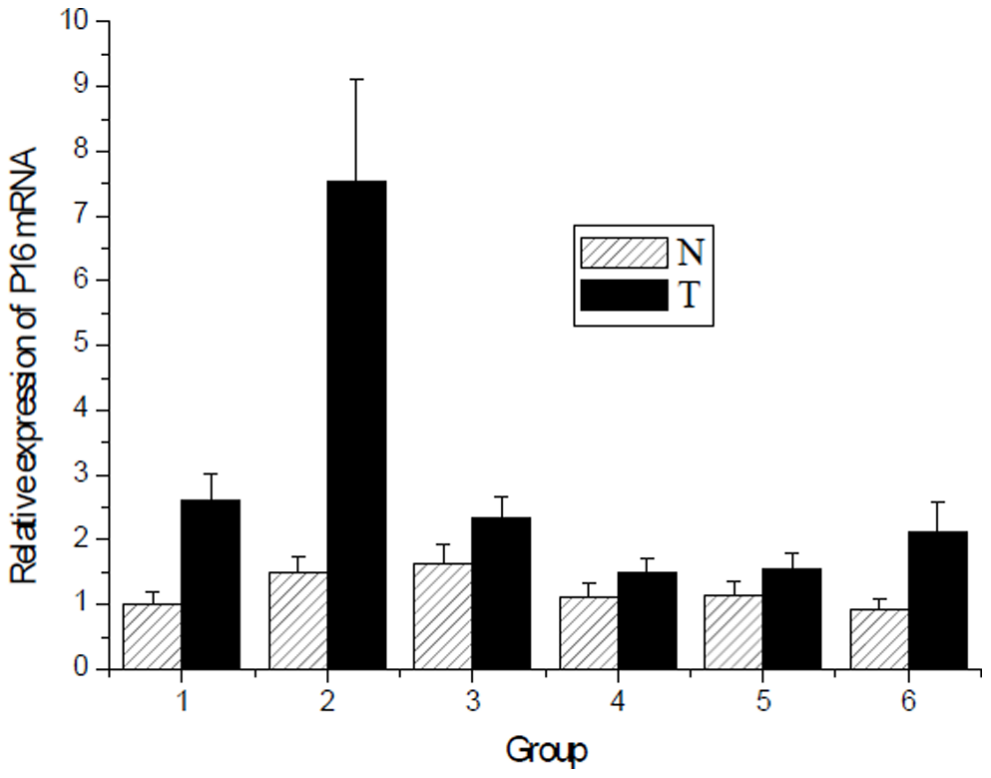
Quantitative RT-PCR analysis of *P*16 mRNA expression in thymic lymphoma tissue samples radiation induced irradiation and normal control non-irradiated thymus tissue samples. N: normal control non-irradiated thymus tissue samples. T: radiation induced thymic lymphoma tissue samples. (T vs N, *P*<0.05).

**Figure 4:**
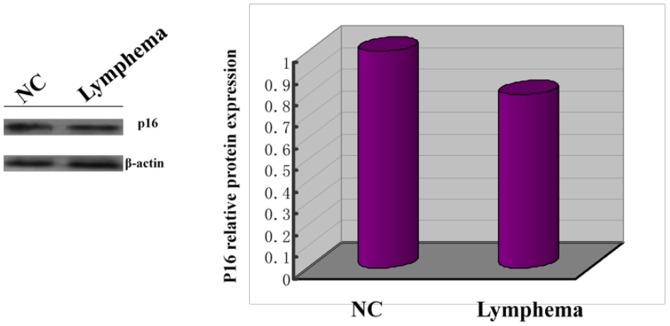
Reduced of *P*16 protein expression in thymic lymphoma induced by irradiation.

The mRNA expression of tumor suppressor gene *p16* in radiation-induced thymic lymphoma tissues and normal non-irradiated thymus tissue samples was examined by qRT-PCR analysis, while the mRNA expression profiles in two groups were analyzed by microarray. Of 45000 mouse genes in the microarray, we found 2876 genes showed significantly different expression between radiation-induced thymic lymphoma samples and normal non-irradiated thymus samples. The expression patterns of *p16* are shown in the G_1_-S pathway (Figure 2), and the important results of microarray were given in Table 1 in detail. We detected the mRNA expression of *p16* in 6 tissue samples from both groups (Figure 3). Moreover, *p16* mRNA expression level was significantly lower in the radiation-induced thymic lymphoma tissue samples compared with matched normal non-irradiated thymus tissue samples. P16 protein expression in both groups was examined by western blot, we found that P16 protein expression level was significantly lower in the radiation-induced thymic lymphoma tissues than that in matched normal non-irradiated thymus tissues (Figure 4).

**Table 1:** Data of mRNA expression with significant difference in G_1_/S cell cycle by the Illumina Sentrix Mouse-6 Expression Bead Chip.

Gene	N.AVG_Signal	T.AVG_Signal	Differnt Score	Differnt Ratio
Cdk4	2480.164	6887.145	63.27042	2.776891
Cdk6	47.29345	220.5876	39.11695	4.664231
Cdkn2b	304.7015	75.08892	−38.45883	0.2464344
Cdkn2d	2720.639	709.1175	−55.01941	0.2606437
Cdk2	3130.801	5331.355	29.05482	1.702872
Atm	300.9656	626.6469	22.72435	2.082121
E2f2	7689.074	3044.281	−36.05586	0.395923
E2f4	1116.279	1935.731	24.09156	1.734093
E2f6	1321.134	2664.024	27.10226	2.016467
Ccnd1	881.3447	2146.841	56.64333	2.43587
Ccnd3	10626.85	4594.292	−29.83043	0.4323286
Tfdp1	4279.905	7409.138	25.3472	1.731146
Pola2	1227.237	2374.903	36.0864	1.935163
Pole	176.3562	360.9415	21.78839	2.046663
Orc5l	1144.603	1950.801	26.35427	1.704348
Orc6l	3755.128	8224.204	35.02106	2.190126
Mcm2	3782.601	6463.503	26.67579	1.708746
Mcm3	1091.692	1804.522	22.5774	1.65296
Mcm6	1852.818	3947.626	24.63652	2.130607

N: normal control non-irradiated thymus tissue samples. T: radiation induced thymic lymphoma tissue samples.

### The methylation pattern in the promoter of the *p16* gene

**Figure 5:**
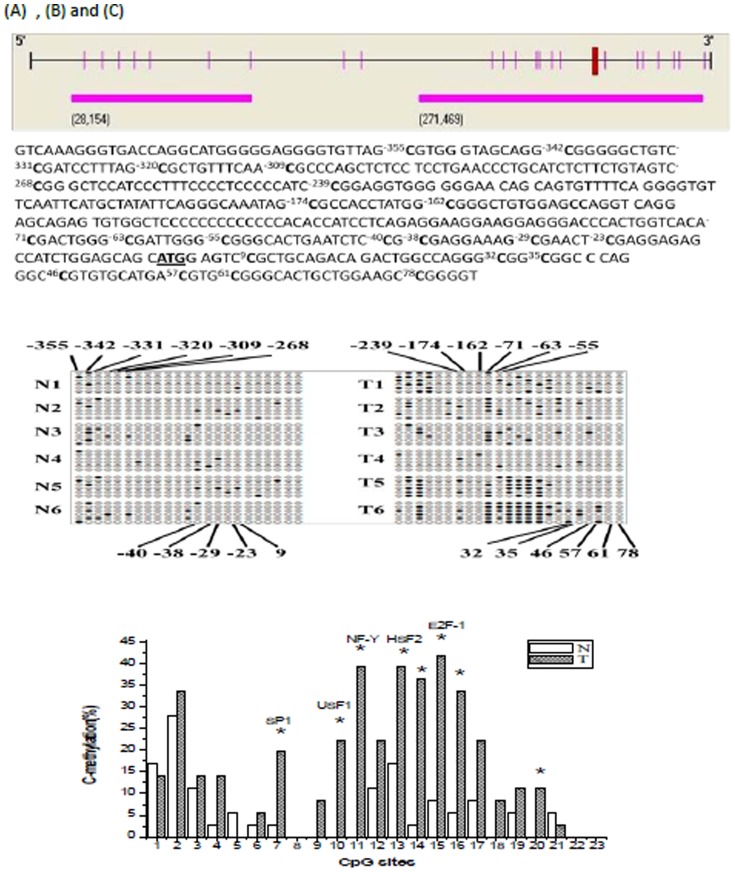
The P16 promoter region and Methylation at the 23 CpG sites. (A) Schematic of the P16 gene promoter region (from 400 nucleotides upstream to 200 nucleotides downstream of the transcription start site) is presented in the upper diagram. The two CpG islands are boxed. The CpG dinucleotides are indicated as |. The major transcription start site is located at −355 upstream to 78 downstream of the ATG translation start site. The 16 CpG sites analyzed for cytosine methylation (bold) with their position relative to the A (chr4: NC_000070, 12509) of the ATG translation start site (underlined) are indicated. (B) Analysis of cloned amplified bisulfite-treated DNA from 6 pairs of radiation induced carcinogenesis tissue samples and normal control non-irradiated thymus tissues. Solid circles are methylated CpG sites. The location of these sites is shown relative to their location in the amplified P16 region. (C) Percent methylation of 16 CpG dinucleotides in the p16 promoter region. CpG sites in Sp1, USF-1, NF-Y, HSF2 and E2F-1 binding sites are indicated. *p<0.05. The numbers 1 to 23 represented 23 methylation sites sequentially (−355 to 78). N: normal control non-irradiated thymus tissue samples. T: radiation induced thymic lymphoma tissue samples.

We also examined the methylation state of CpG islands in the *p16* promoter. Two CpG islands were found from 400 nucleotides upstream to 200 nucleotides downstream of the *p16* TSS (Figure 5A). The CpG islands were located from nucleotides −355 to78 (relative to the A of the ATG translation start site). We identified 23 CpG dinucleotides located at nucleotides of −355, −342, −331, −320, −309, −268, −239, −174, −162, −71, −63, −55, −40, −38, −29, −23, 9, 32, 35, 46, 57, 61 and 78 in the *p16* gene promoter. To test the methylation state of a CpG island in the *p16* promoter, DNA samples from both groups were treated with bisulfite to convert unmethylated cytosines to uracil and leave methylated cytosines unmodified. The region containing two CpG islands was amplified by PCR, cloned into T vector, and sequenced. The methylation pattern of CpG islands in the *p16* promoter was shown in Figure 5B. By using the Chi-square test, we found that the methylation percentages of −71, −63, −239, −29, −38, −40, −23, 46 CpG sites were significantly higher in radiation-induced thymic lymphoma tissue samples compared with matched control non-irradiated thymus tissue samples (P<0.05, P<0.01, Figure 5). The highest methylation was at the −29 CpG site.

## Discussion

Ionizing-radiation-induced leukemogenesis and lymphomagenesis is a complex process involving both genetic and epigenetic changes [24]. Ionizing radiation induces genomic instability, which is mainly characterized as cell necrosis, chromosomal aberration, increased apoptosis, micronucleus formation, changes in gene expression, and abnormal DNA methylation [25], [26]. Currently, the genetic changes have been clarified by several studies, whereas the studies on epigenetic changes have recently been carried out due to the limitations in detection technology. DNA methylation is an important part of epigenetics. Extensive genomic hypomethylation, hypomethylation of CpG islands in the promoter region of oncogenes, and hypermethylation of CpG islands in the promoter region of the tumor suppressor genes are frequent in hematological malignancies caused by radiotherapy, or in other tumors. It has been suggested that the change in DNA methylation status plays an important role in the process of tumor occurrence and development [27].

The fate of cells is tightly controlled by a series of cytokines, of which CDK4 is the key enzyme controlling the process of G_1_ phase. CDK4 activity is regulated by P16 protein encoded by *p16* gene. P16 protein prevents cell S phase entry by specifically inhibiting CDK4 and inducing cell cycle arrest, thus *p16* acts as a tumor suppressor gene. Once P16 is inactivated, cell growth is accelerated and proliferation becomes abnormal, eventually leading to tumorigenesis [28]. Methylation of CpG islands in the promoter region of tumor suppressor genes is one of the important factors for the inactivation of tumor suppressor genes. It is involved in the occurrence and development of a variety of tumors [29]. Previous studies revealed that *p16* gene hypermethylation plays an important role in the development of a variety of tumors [30]–[32]. The changes in *p16* gene methylation status in radiation-induced tumorigenesis have not widely been investigated, although the changes in *p16* gene methylation status may be important in the process of ionizing-radiation-induced tumorigenesis. In contrast, Kovalchuk et al. showed that methylation in the promoter of *p16* was increased significantly in the normal liver tissues of mice exposed acute and chronic low dose irradiation [33]. Our study tended to find the changes of every single CpG point in the promoter of *p16* in the process of ionizing-radiation-induced tumorigenesis. When the methylation rate is increased, the affinity of the gene-transcription-factor binding sites for the cognate transcription factor is reduced, thus the gene transcription is inhibited and the protein expression level is decreased [34].

Our study showed that 6 months after irradiation, *p16* mRNA and protein levels in the mouse thymocytes were significantly inhibited compared with control group. P16 protein expression levels in thymic lymphoma cells were downregulated. Twenty-three CpG sites of the CpG islands in the promoter region of *p16* gene were identified. DNA methylation at −71, −63, −239, −29, −38, −40, −23 and 46 CpG sites in thymic lymphoma cells was significantly increased after irradiation, but there was no significant change at other sites. DNA sequence analysis using TESS software showed that the −239, −40, −71, −63 and −38 CpG sites corresponded to the binding sites for transcription factors SP1, HSF2, USF1, NF-Y and E2F- 1, respectively. Increased methylation at the −239, −40, −71, −63 and −38 sites in the *p16* promoter region may inhibit the binding of corresponding transcription factors to the transcription factor binding sites of the promoter region, and then down regulate *p16* gene and protein expression. Thus, the inhibitory effect of P16 protein on the cell cycle is reduced and cell proliferation is uncontrolled, and thus eventually leads to tumorigenesis. The *p16* promoter region CpG islands hypermethylation of mouse liver tissue could be induced by low dose whole body irradiation [33]. At the same time, in many studies of radiation, hypermethylation of promoter region and hypomethylationof global DNA could be induced by irradiation in many cell lines including normal cell lines [35]–[37]. These studies provided important information on alterations in DNA methylation as one of the determinants of radiation effects, which may be associated with altered gene expression, especially *p16* gene. Since alterations in DNA methylation have also emerged as one of the most consistent molecular alterations in cancer, these data also suggest the possibility that radiation-induced carcinogenic risk might be affected by complicated DNA methylation. Combined with the results, we concluded that the *p16* promoter region CpG islands hypermethylation is one of the important epigenetic changes in tumorigenesis of mouse thymus caused by radiation. However, further studies are needed to analyse and detect the initiation factors under the influence of above five CpG sites, and to explore the effects of CpG island hypermethylation in the *p16* gene promoter region on ionizing-radiation-induced tumorigenesis.
